# Are people at high risk for diabetes visiting health facility for confirmation of diagnosis? A population-based study from rural India

**DOI:** 10.1080/16549716.2017.1416744

**Published:** 2018-01-15

**Authors:** Nikhil Srinivasapura Venkateshmurthy, Kathirvel Soundappan, Balaji Gummidi, Malipeddi Bhaskara Rao, Nikhil Tandon, K. Srinath Reddy, Dorairaj Prabhakaran, Sailesh Mohan

**Affiliations:** ^a^ Public Health Foundation of India, Gurgaon, India; ^b^ Department of Community Medicine, School of Public Health, Post Graduate Institute for Medical Education and Research, Chandigarh, India; ^c^ MyCure Hospitals, Visakhapatnam, India; ^d^ Department of Endocrinology and Metabolism, All India Institute of Medical Sciences, New Delhi, India; ^e^ Centre for Chronic Disease Control, Gurgaon, India

**Keywords:** Confirmatory test, diabetes mellitus, screening, SORT IT, UDAY

## Abstract

**Background**: India is witnessing a rising burden of type 2 diabetes mellitus. India’s National Programme for Prevention and Control of Diabetes, Cancer, Cardiovascular diseases and Stroke recommends population-based screening and referral to primary health centre for diagnosis confirmation and treatment initiation. However, little is known about uptake of confirmatory tests among screen positives.

**Objective**: To estimate the uptake of confirmatory tests and identify the reasons for not undergoing confirmation by those at high risk for developing diabetes.

**Methods**: We analysed data collected under project UDAY, a comprehensive diabetes and hypertension prevention and management programme, being implemented in rural Andhra Pradesh, India. Under UDAY, population-based screening for diabetes was carried out by project health workers using a diabetes risk score and capillary blood glucose test. Participants at high risk for diabetes were asked to undergo confirmatory tests. On follow-up visit, health workers assessed if the participant had undergone confirmation and ask for reasons if not so.

**Results**: Of the 35,475 eligible adults screened between April 2015 and August 2016, 10,960 (31%) were determined to be at high risk. Among those at high risk, 9670 (88%) were followed up, and of those, only 616 (6%) underwent confirmation. Of those who underwent confirmation, ‘lack of symptoms of diabetes warranting visit to health facility’ (52%) and ‘being at high risk was not necessary enough to visit’ (41%) were the most commonly reported reasons for non-confirmation. Inconvenient facility time (4.4%), no nearby facility (3.2%), un-affordability (2.2%) and long waiting time (1.6%) were the common health system-related factors that affected the uptake of the confirmatory test.

**Conclusion**: Confirmation of diabetes was abysmally low in the study population. Low uptake of the confirmatory test might be due to low ‘risk perception’. The uptake can be increased by improving the population risk perception through individual and/or community-focused risk communication interventions.

## Background

Diabetes is an important non-communicable disease (NCD) in India, which is home to the second largest number of people with diabetes, i.e. 69 million. This number is estimated to increase to 140 million by 2040 [1]. The prevalence of diabetes in India is 7.3% [2], and the burden of undiagnosed diabetes is also high. Estimates suggest that almost 50% of those with the disease are undiagnosed [1].

Diabetes satisfies most of the criteria for screening [3]. A number of institutions recommend screening for diabetes [4–7]. The National Programme for Prevention and Control of Cancer, Diabetes, Cardiovascular diseases and Stroke (NPCDCS) also recommends screening for diabetes together with other NCDs like hypertension and common cancers (oral, breast and cervical) [8]. The Government of India started NPCDCS in 2008. It is currently being implemented in 468 districts. Apart from screening, the NPCDCS recommends identifying and addressing modifiable risk factors, diagnosis of diabetes based on protocols and follow-up at the community level.

Under NPCDCS, the Accredited Social Health Activists (ASHA) are entrusted with the responsibility of risk assessment of all adults aged ≥30 years in their service area using a risk score [8]. The risk is assessed based on age, use of tobacco and alcohol, waist circumference, physical activity and family history of diabetes, hypertension and heart disease. A person scoring more than four on the risk score is determined to be at high risk and is encouraged to participate in the screening camp to be organized at the village/sub-centre. The auxiliary nurse midwife (ANM) assisted by ASHA will screen for diabetes. Those with a random blood sugar of 140 mg/dl and above are to be referred to a medical officer, at the nearest facility, for confirmation of diagnosis and initiation of treatment.

It is crucial that those who are at high risk and undiagnosed are identified through screening. The screen positives are required to visit the health facility for confirmation of diagnosis. If the confirmation of diagnosis does not happen, the programme will fail to effectively address the growing burden of diabetes. Only a limited number of studies on confirmation of diabetes among screen positives have been published from India [9–11]. We analysed data from an ongoing community-based study to determine the socio-demographic, behavioural and clinical factors of population screened for diabetes, uptake of the confirmatory test for diabetes and the reasons for non-uptake.

## Methods

### Study setting

Andhra Pradesh is situated in the southern part of India. The prevalence of diabetes in Andhra Pradesh is 8.4% [4]. Visakhapatnam is the fourth largest district of Andhra Pradesh. Makavarapalem and Nathavaram are two mandals (administrative units under a district) in Visakhapatnam district. The combined population of these two mandals is 118,659 with 59,540 adults aged ≥30 years. Four primary health centres and 22 health sub-centres deliver the health services under the public health system in these mandals.

### Project UDAY

**Figure 1.**
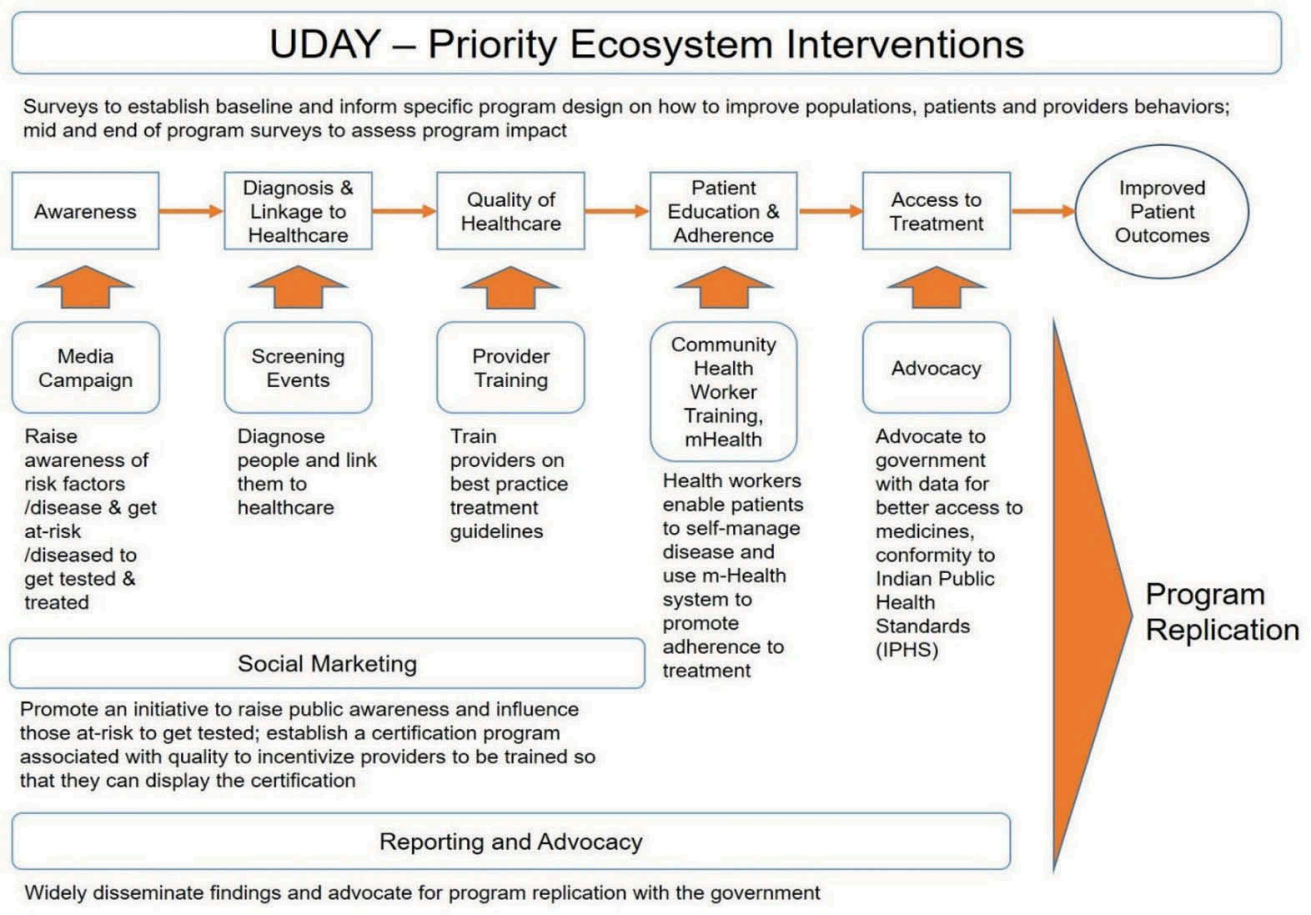
Implementation framework and components of UDAY.

Since 2013, Public Health Foundation of India (along with partners Population Services International and Project Hope) has been implementing project UDAY in these two mandals. The components of project UDAY are summarized in the framework below (Figure 1). UDAY is a comprehensive diabetes and hypertension prevention and management programme. Among many other components, screening of adults aged ≥30 years for diabetes is an important component of UDAY. A total of 22 trained health workers of UDAY are engaged in screening. The health workers visit the households and screen all eligible adults. They collect information on socio-demographic factors, use of tobacco and alcohol in the past 30 days, family history of any cardio-metabolic diseases (diabetes, hypertension, cardiovascular disease or stroke) and date and time of last meal. They measure blood pressure, height, weight and waist circumference using standard procedures. Using a glucometer, they measure capillary blood glucose. Based on the difference between the time of the capillary blood glucose test and the time of the last meal, the blood glucose value was either determined as fasting (difference of ≥8 hours) or random (difference <8 hours).

An android-based application has been specially designed for screening. Each participant is assigned a unique participant ID (PID) by the application. The application incorporates a validated diabetes risk score [12] to identify those at high risk. After the health worker enters the information, the application calculates risk score based on four parameters – age, family history of diabetes, blood pressure and waist circumference. Those scoring more than 16 are determined to be at high risk. Apart from the score, those with a fasting capillary blood glucose of ≥126 mg/dl or random capillary blood glucose of ≥200 mg/dl were also classified as high risk. The high-risk individuals are counselled by the health workers to visit the nearby health facility to get the diabetes diagnosis confirmed. They also educate these individuals about the risk status and steps to be taken to modify their lifestyle for risk reduction.

After a period of approximately two months, the health worker visits the household of those at high risk for a follow-up. In this follow-up visit, the health worker records if the high-risk individual has visited the health facility and the status of the diagnosis. If the individual did not visit the health facility, the reasons for this are elicited. The health worker also reminds the individual about her/his risk status and the benefits of early diagnosis and initiation of treatment. S/he motivates the individual to visit the health facility. The information collected during the follow-up visit is entered into a specially designed Android application. The PID generated during the screening is used during the follow-up, to avoid duplication and maintain seamless workflow.

Mass media, mid-media and inter-personal communication (IPC) activities were implemented by the Population Services International (PSI). The mass media campaign consisted of advertisements in the local television channels, hoardings and wall paintings; the mid-media campaign consisted of street theatre activity; the IPC campaign consisted of one-to-one and one-to-many sessions by trained IPC coordinators. All three communication activities focused on current lifestyle; increasing burden of diabetes in the community; the importance of getting screened for diabetes and adoption of healthy lifestyle (increased consumption of fruits and vegetables, avoiding high-calorie food, increasing physical activity); screening and follow-up activity under UDAY.

### Study design

We analysed the screening and follow-up activity data collected under project UDAY. The screening for diabetes was started in April 2015. The follow-up was initiated in June 2015. Both screening and follow-up are ongoing. For the present study, screening and follow-up data collected between April 2015 and August 2016 were analysed.

### Data source and data analysis

The data were retrieved from the central server placed at PHFI, Gurgaon. The screening and follow-up data were merged using the unique PID. Variables were summarized using means (standard deviation) or frequencies (percentages). The proportion of those who underwent confirmation was calculated. The number (percentage) of reasons for not undergoing confirmation was estimated. Data management and analysis were carried out in Stata (StataCorp. 2015. Stata Statistical Software: Release 14. College Station, TX: StataCorp LP.).

### Ethics approval

Permission was obtained from the Institutional Ethics Committee, Public Health Foundation of India, Gurgaon, India and the Ethics Advisory Group of the International Union Against Tuberculosis and Lung Disease, Paris, France for analysis of the data.

## Results

**Figure 2.**
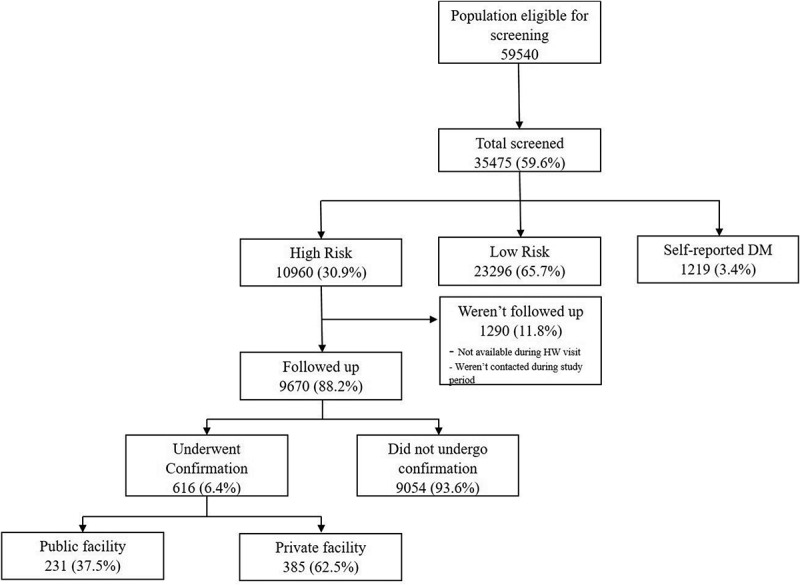
Flow chart depicting the population screened for diabetes and followed up in Makavarapalem and Nathavaram mandals, Visakhapatnam India between April 2015 and August 2016.

Out of the 59,540 persons eligible for screening, 60% were screened in the duration between April 2015 and August 2016 (Figure 2). The mean (±SD) age of the screened population was 48.2 (±13.0) years, and 60% were females. The fasting capillary blood glucose was measured among 4345 (12.3%) participants and random capillary blood glucose among 31,065 (87.8%). The mean (±SD) fasting capillary blood glucose was 96.3 (±28.5) mg/dl, and the mean random capillary blood glucose was 114.9 (±44.7) mg/dl. Nearly 31% of the screened population was determined to be at high risk for developing diabetes (Table 1). The proportion of participants with high risk was highest among participants with a family history of CVD.

**Table 1.** Socio-demographic, and behavioural factors of eligible population (≥30 years) screened in Makavarapalem and Nathavaram mandals between April 2015 and August 2016.

Characteristics	Total (%)^a^		High risk (%)^b^		Low risk (%)^b^		Self-reported DM (%)^b^	
*N*	35,475	(100)	10,960	(30.9)	23,296	(65.7)	1219	(3.4)
Age (years)								
30–39	10,752	(30.3)	1789	(16.6)	8800	(81.8)	83	(0.8)
40–49	8753	(24.7)	2986	(34.1)	5484	(62.7)	283	(3.2)
50–59	6863	(19.3)	1763	(25.7)	3757	(54.7)	343	(5.0)
≥60	9107	(25.7)	3422	(37.6)	5175	(56.8)	510	(5.6)
Sex								
Male	14,108	(39.8)	4912	(34.8)	8539	(60.5)	657	(4.7)
Female	21,367	(60.2)	6048	(28.3)	14,757	(69.1)	562	(2.6)
Family history of DM								
Yes	2007	(5.7)	906	(45.1)	826	(41.2)	275	(13.7)
No	33,468	(94.3)	10,054	(30.0)	22,470	(67.1)	944	(2.8)
Family history of HTN								
Yes	4169	(11.8)	1739	(41.7)	2236	(53.6)	194	(4.7)
No	31,306	(88.2)	9221	(29.5)	21,060	(67.3)	1025	(3.3)
Family history of CVD								
Yes	170	(0.5)	82	(48.2)	77	(45.3)	11	(6.5)
No	35,305	(99.5)	10,878	(30.8)	23,219	(65.8)	1208	(3.4)
Family history of Stroke								
Yes	481	(1.4)	214	(44.5)	241	(50.1)	26	(5.4)
No	34,994	(98.6)	10,746	(30.7)	23,055	(65.9)	1193	(3.4)
Tobacco use								
Yes	12,618	(35.6)	4308	(34.1)	7986	(63.3)	324	(2.6)
No	22,857	(64.4)	6652	(29.1)	15,310	(67.0)	895	(3.9)
Alcohol use								
Yes	6224	(17.5)	2287	(36.7)	3737	(60.0)	200	(3.2)
No	29,251	(82.5)	8673	(29.7)	19,559	(66.9)	1019	(3.5)
Mean (SD) SBP^c^	125.7	(21.7)	138.9	(20.3)	118.7	(18.9)	139.4	(22.0)
Mean (SD) DBP^c^	76.0	(11.7)	83.2	(11.2)	72.4	(10.2)	80.6	(11.6)
Mean (SD) Fasting capillary glucose (mg/dl)^d^	96.3	(28.5)	101.9	(36.2)	90.8	(12.7)	159.8	(70.9)
Mean (SD) Random capillary glucose (mg/dl)^e^	114.9	(44.7)	118.9	(47.8)	107.9	(29.3)	213.9	(99.9)
Mean (SD) waist circumference (cm)^f^	77.1	(11.8)	83.5	(11.1)	73.5	(10.4)	88.0	(11.8)

^a^Column percentage; ^b^row percentage; DM – diabetes mellitus; HTN – hypertension; CVD – cardiovascular disease; SBP – systolic blood pressure; DBP – diastolic blood pressure; SD – standard deviation; ^c^data missing for five participants; ^d^data missing for eight participants; ^e^data missing for 57 participants; ^f^data missing for 368 participants. Participants scoring >16 on risk score classified as high risk.

Among those at high risk, nearly 88% were followed up by health workers in the period between April 2015 and August 2016. Of the remainder, some were not present when health workers visited the household, and others were planned to be followed up after August 2016 (data not presented). Only 616 (6.4%) visited any health facility to undergo confirmation. Three hundred and eighty-five (62.5%) out of 616 visited a private health facility. The proportion of the high-risk population visiting health facility for confirmation of diagnosis was lowest among those in the age group of 30–39 years (Table 2). The high-risk population with a positive family history of diabetes, cardiovascular disease and stroke were more likely to get the confirmation of diagnosis done.

**Table 2.** Comparison of socio-demographic, clinical and behavioural factors between those who underwent the confirmatory test and those who did not in Makavarapalem and Nathavaram mandals between April 2015 and August 2016.

Characteristics	Underwent confirmation (%)^a^		Did not undergo confirmation (%)^a^	
*N*	616	(6.4)	9054	(93.6)
Age (years)				
30–39	64	(4.2)	1456	(95.8)
40–49	164	(6.2)	2467	(93.8)
50–59	175	(7.0)	2309	(93.0)
≥60	213	(7.0)	2822	(93.0)
Sex				
Male	260	(6.0)	4076	(94.0)
Female	356	(6.7)	4978	(93.3)
Family history of DM				
Yes	66	(8.1)	748	(91.9)
No	550	(6.2)	8306	(93.8)
Family history of HTN				
Yes	90	(5.8)	1472	(94.2)
No	526	(6.5)	7582	(93.5)
Family history of CVD				
Yes	6	(9.0)	61	(91.0)
No	610	(6.4)	8993	(93.6)
Family history of stroke				
Yes	18	(9.5)	172	(90.5)
No	598	(6.3)	8882	(93.7)
Tobacco use				
Yes	232	(6.1)	3555	(93.9)
No	384	(6.5)	5499	(93.5)
Alcohol use				
Yes	95	(4.7)	1906	(95.3)
No	521	(6.8)	7148	(93.2)
HTN (SBP ≥ 140 or DBP ≥ 90)				
Yes	365	(7.8)	4341	(92.2)
No	251	(5.1)	4713	(94.9)
WC (>90 cm in males or >80 in females)^b^				
Yes	294	(6.3)	4394	(93.7)
No	321	(6.6)	4568	(93.4)

^a^Row percentage; DM – diabetes mellitus; HTN – hypertension; CVD – cardiovascular disease; SBP – systolic blood pressure; DBP – diastolic blood pressure; SD – standard deviation; ^b^data missing for 93 participants.

**Figure 3.**
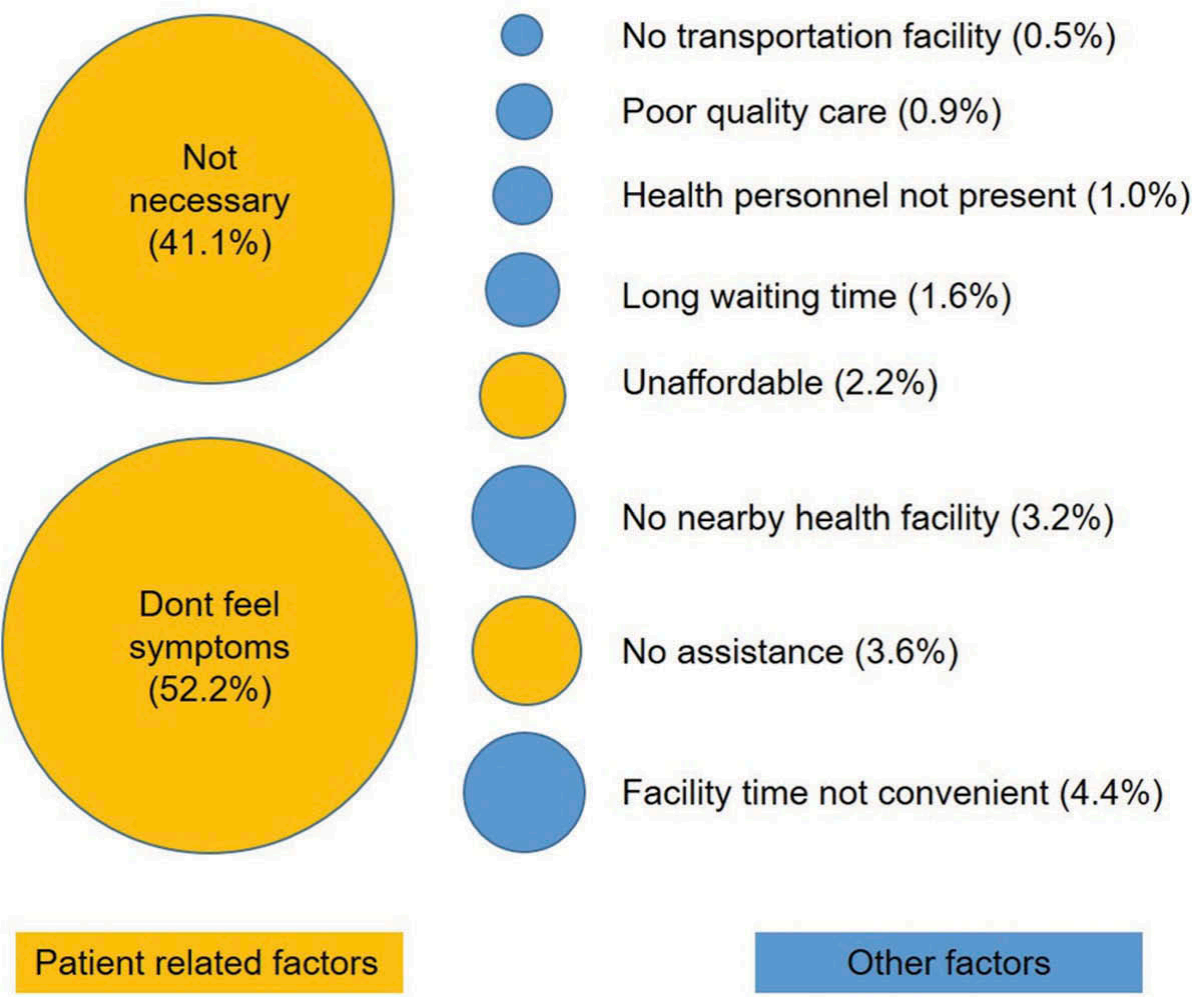
Reasons for not getting the confirmation of diabetes stated by participants screened and followed up between April 2015 and August 2016.

Health workers asked those who did not seek confirmation to list the reasons for not doing so. The majority of them reported a ‘lack of symptoms of diabetes warranting visit to health facility’ (52.2%) and ‘being at high risk was not necessary enough to visit’ (41.1%) as the main reasons. Few also reported health system-related (‘facility time not convenient’) and support-related (‘no assistance’) factors as the reasons (Figure 3).

## Discussion

This is the first study from rural India to assess the uptake of the confirmatory test for diabetes by those at high risk after undergoing population-based screening. Only 6% of those at high risk underwent confirmation. The majority chose to visit the private health facility. Those who did not seek confirmation cited a lack of any symptoms and no necessity to visit a health facility as the major reasons for the same. We do not report bivariate and multivariate analyses to test the associations between various socio-demographic, clinical and biological factors and not undergoing confirmation for diabetes. Even though the measure of association was statistically significant for age group, alcohol use and high blood pressure, the interpretation was not relevant, given the high numbers in the high-risk population and the low numbers of those who underwent confirmation.

Studies from India report varied estimates of confirmation of diabetes post screening. Two studies report percentages of 50% and 30% respectively [9,11]. The higher percentage is because the screening for diabetes and confirmation were carried out in the hospital setting. Another study that employed a population screening approach reported that <1% underwent confirmation [10]. The number screened and at high risk was substantially higher in our study than the three mentioned above. This may be due to the fact that the screening was community-based and used a risk score.

We attribute the low rate of confirmation of diabetes to the low ‘risk perception’ in the screened community. Risk perception combines subjective judgements and evaluations of one’s potential hazards [13]. The low risk perception is because a layperson finds it difficult to understand his/her risk status in the absence of any somatic experience or disease symptom. A disease symptom (e.g. fever in case of malaria) drives action (visiting a health facility for diagnosis and treatment) rather than an abstract concept of risk in the absence of symptoms. Studies report that increasing age, low literacy levels and pre-existing risk factors contribute to a low risk perception [14,15]. The Health Belief Model states that the following six constructs – perceived susceptibility, perceived severity, perceived benefits, perceived barriers, cues to action and self-efficacy – influence an individual’s decision to take action [16]. An individual is more likely to take action if he or she perceives himself or herself to be at risk of getting a serious disease and believes that he or she can control a particular behaviour. Tailoring the risk information to an individual’s characteristics and behaviours and specifying the consequences of inaction and recommended action may help in improving the risk perception [17]. Training of those communicating risk and framing the message to make it easy to understand are some of the ways that have been suggested to improve risk perception [18,19].

Among those who underwent confirmation by visiting a health facility, nearly 65% chose a private health facility. It is estimated that nearly 80% of primary care visits in rural India are to a private health facility, even when a qualified doctor provides a free service through a public health facility [20]. This may be due to perceived deficiencies in the public health facilities or better service provision by the private health facilities. A systematic review of performance of public and private healthcare systems in low- and middle-income countries reports that public health facilities lack timeliness and hospitality towards patients [21]. The proportion visiting a private health facility in our study is very high, considering the fact that the cost of care in private facilities is four times higher than in public [22] counterparts. This leads to huge out-of-pocket and catastrophic health care expenditure.

This study has many strengths. It is the first study to report results of confirmation of diabetes diagnosis from rural India and in a large free-living population. The study employed population-based screening strategy, which is also suggested by the NPCDCS. Trained health workers used an android-based application to collect information and generate risk scores. This helped in real-time data collection and monitoring. The logic checks and mandatory fields in the application resulted in very few errors and missing data. We do, however, acknowledge a few limitations. Our study could have been stronger if the reasons for not undergoing confirmation were explored in more depth through qualitative research. This would have shed more light on the contextual factors and help to understand the underlying factors better. Though a qualitative study was planned, we could not carry out due to reasons beyond the investigators’ control. Since the project is still being implemented, we are not able to report the screening yield.

The results have had implications for implementation of UDAY. The findings have helped the programme manager to rethink the strategy to tailor and contextualize communication of risk to high-risk participants. We discussed the results with health workers together with their supervisors and asked them to come up with solutions to improve the confirmation of diabetes diagnosis. They suggested the use of flipcharts with pictures and minimal text to be used during the interaction with high-risk participants. The flipcharts were designed with the inputs of health workers, and the health workers were trained to use them in the field. During the subsequent meetings with health workers, we have received positive feedback about the strategy, and uptake of the confirmatory test has picked up.

The findings are relevant to the NPCDCS as well. The guidelines suggest population-based screening for NCDs including diabetes and referral to primary health centre for confirmation. If the risk is not communicated properly by the ASHA or ANM, the screen positives may not seek confirmation. Studies report that perceived risk is always lower than actual risk [23]. Since NCDs in general and diabetes in particular require lifestyle modifications that are difficult to initiate and maintain, people might not like to know about their disease status or postpone knowing their status. This would lead to an increased burden of undiagnosed cases and hinder efforts to address the growing burden.

## Conclusion

The rate of confirmation of diagnosis of diabetes among the high-risk population was low. Most high-risk participants visited a private health facility for confirmation of diagnosis. The low confirmation is due to low risk perception. Effective communication of risk is needed to improve the rate of confirmation.

## Paper context

The burden of diabetes is high in India. Guidelines recommend screening for diabetes. Evidence on uptake of confirmation of diagnosis post screening is lacking. We report the results of community-based screening under UDAY between April 2015 and August 2016. Only 6% of screen positives underwent confirmation. The majority visited a private health facility. Improving the risk perception through effective communication is crucial to increase the confirmation of diagnosis and key to the success of national programme.
